# Current trends and perspectives in the exploration of anorexia athletica-clinical challenges and therapeutic considerations

**DOI:** 10.3389/fnut.2023.1214398

**Published:** 2023-07-17

**Authors:** Octavian Vasiliu

**Affiliations:** Department of Psychiatry, Dr. Carol Davila University Emergency Central Military Hospital, Bucharest, Romania

**Keywords:** anorexia athletica, anorexia nervosa, ideal body image, eating disorders, elite athletes, sports psychiatry, esthetic sports

## Abstract

Elite athletes are exposed to a considerable amount of physical and psychological stress throughout their entire professional life, but the exploration of the consequences of this stressful regimen on mental health is still in its early stages. Eating disorders (EDs), substance use disorders, and behavioral addictions represent only several domains that are worth more investigation in this vulnerable population, in order to find preventative and therapeutic strategies. The exploration of EDs in athletes is important because this population is very vulnerable to the impact that weight and body shape may have on their professional performances, and epidemiological studies support this concern, i.e., the prevalence of EDs in athletes is significantly higher than in the general population. This article is dedicated to the synthesis of available data regarding a specific pathology reported in elite athletes, i.e., anorexia athletica (AA), based on a narrative reviewing methodology. The information about risk factors, pathophysiology, positive and differential diagnosis, epidemiology, structured evaluation, and treatment of AA have been summarized and future research directions have been highlighted. While tentative diagnostic criteria for AA and a self-administered questionnaire exist, its pathophysiology is still insufficiently explored, and the treatment is not based on good-quality trials. According to the retrieved data, more research targeting the physical and mental health of elite athletes, especially those practicing esthetic sports, is needed, in order to implement adequate screening and early intervention programs. Future studies targeting various sub-populations of elite athletes, i.e., esthetic vs. non-esthetic sports, individuals presenting a history of ED vs. those without such a history, and those practicing individual sports vs. team sports are needed to reach the objective of improving the quality of life in this population.

## 1. Introduction

The exploration of specific psychopathological aspects in athletes has a relatively short history, although the impact of such an investigative process is expected to present significant positive consequences on the quality of life in this population (1–4). Low body weight has been traditionally considered an important quality in several sports activities, possibly leading to advantages in a direct confrontation with other athletes (5). The onset of different types of anorexia [i.e., with/without binge eating/purging pattern, with/without restrictive eating or excessive exercising, with normal (recovering), significantly or dangerously low body weight, and other specified/unspecified anorexia nervosa] (6, 7) may be the result of the external pressure for a lean figure and internal striving for thinness in elite athletes practicing esthetic sports. In these individuals, an obsessive focus on low body weight, combined with a severe decrease in calorie intake and/or excessive involvement in physical exercise, aimed at decreasing or preserving low body mass index (BMI), suggests the onset of anorexia athletica (AA) (5). However, it is important to take into consideration the possibility of low body weight and decreased fat mass distribution in athletes due to their basic training, and not secondary to the AA onset; therefore, integrating these variables in a larger context, using other non-anthropometric parameters, is necessary (5).

The prevalence of eating disorders (EDs) among elite athletes was higher than in the general population, according to an epidemiological study in Hungary (8). Although the average BMI was in the normal range, high rates of anorexia nervosa (AN) and bulimia nervosa (BN) were detected, i.e., 16.7 and 6.9%, respectively, in athletes (8). These findings were confirmed in a two-phase study, including self-reports and clinical interviews, that enrolled Norwegian elite sports students (*N* = 677) and controls (*N* = 421) (9). EDs prevalence was higher in the study group (7 vs. 2.3%) and in females vs. male athletes (14 vs. 3.2%) (9).

Anorexia athletica or the “female athlete triad” was described for the first time by the American College of Sports Medicine and updated in 2007 (10–12). The core elements of AA were dysfunctions of energy availability (i.e., low energy resources), menstrual cycle, and bone mineral density (10, 13). The concept of AA is not unanimously accepted, and there are authors who consider the distinction between AN and AA as “unreasonable,” based on the potentially common pathogenetic background and on the need to consider all EDs in a dimensional paradigm (14). The diagnosis of AA is not formally recognized by the current diagnostic systems, i.e., the 5th edition of the Diagnostic and Statistical Manual of Mental Disorders, text revised (DSM-5TR) and the 11th edition of the International Classification of Diseases (ICD-11) (6, 7).

Starting from (1) the high rate of EDs in athletes, reported in the literature, (2) the apparent contradiction in the description of AA as an independent disorder, and (3) the necessity to improve the quality of life and prognosis of athletes diagnosed with any type of eating disorder, the aim of this article is to explore the data available to support the existence of AA and to define potential future directions for research. In order to retrieve relevant information for the aim of the current article, a narrative reviewing methodology was applied, consisting in searching three electronic databases, i.e., PubMed—https://pubmed.ncbi.nlm.nih.gov/, Cochrane—https://www.cochrane.org/, and Google Scholar—https://scholar.google.com/, for papers published up to March 2023 (with no inferior time limit) and consulting all significant references of the reviewed articles. The search paradigm was “anorexia athletica” OR “female athlete triad” OR “eating disorders” AND “sport” OR “athletes” OR “gymnasts” OR “Sports psychiatry” OR “Sports medicine.” Both primary and secondary reports have been reviewed.

## 2. Synthesis of the available data regarding anorexia athletica

The weight-related burden on female athletes could be higher than for men due to the two directions of pressure on physical leanness: (1) the demand to be thin as a general sociocultural demand for women and (2) the more specific request for leanness as a pre-condition for better professional performances (15). For athletes like distance runners, sprinters, or swimmers, low body weight is conceived as a competitive advantage, while for gymnasts, figure skaters, or ballerinas, low BMI is associated with both better performance and appearance, as determined by judges (15–17). Female athletes in endurance or appearance sports are exposed to a higher risk for eating disorders (16).

### 2.1. Risk factors and pathophysiology

The risk factors, as well as the pathophysiological elements, can be distributed in two large categories, i.e., psycho-social (e.g., self-perceived body image, idealized body image and shape, personal medical and psychiatric history, negative emotional experiences related to sports activity, and personality factors) and biological (e.g., leptin, adiponectin, or estrogen levels). These factors need to be interpreted in relation to the specific profile of each sports activity (for example, esthetic sports, weight-class sports, or gravitational sports) which raises particular challenges, e.g., the body weight above a certain limit prevents the athlete to compete in a certain wrestling event, while the body shape and weight may offer certain advantages in competing with others in gymnastics (18, 19). Also, it should be noted that retrieved data about the risk factors and pathophysiology of AA have to be extrapolated mainly from studies with other different EDs, because studies focused strictly on AA are very few.

In ballet dancers, the BMI was higher vs. AN patients but lower than in the healthy controls (20). The ED-related psychopathology was lower in ballet dancers, and the self-concept was more positive than in AN patients (20).

A large populational study published in 2004 enrolled all Norwegian elite athletes and healthy controls to detect the risk of presenting EDs, and concluded that individuals practicing leanness- and weight-dependent sports had a higher incidence of these disorders (21). In another study (*N* = 117 at-risk for ED female athletes), conducted by the same Norwegian researcher, the vast majority of participants (*N* = 92) met the criteria for AN, BN, or AA (22). The criteria for AA used in this study were (1) mandatory—weight loss (more than 50% of the expected body weight), gastrointestinal complaints, absence of medical illness, or affective disorder able to explain the weight reduction, excessive fear of becoming obese, and restriction of caloric intake, and (2) optional—delayed puberty, menstrual dysfunction, disturbance of body image, use of purging methods, binge eating, or compulsive exercising (22). Compared to controls (*N* = 30 athletes randomly chosen, considered not at risk for EDs), athletes with EDs had a long history of sports-specific training and dieting (22). Risk factors for EDs in this group were considered longer diets, more numerous weight variations, fast increases in the exercise schedule, and emotionally negative sport-related experiences (22).

Other authors consider the onset of AA dependent on the paradigm of physical appearance in sports, the relation between performance and weight, and sociocultural pressures for thinness or idealized body weight (23). The effect of stressors in adolescents with vulnerable personalities has been associated with restrictive dieting and serotonin metabolic dysfunctions, especially in females (14). Idealized body images and changes in the perception of body shape and weight are also considered important pathogenic factors in EDs, including in the case of athletes (14). The most vulnerable athletes for EDs onset are performing in sports where the body size/shape is valued, a higher power/weight ratio is required, or where weight categories are used (23). Personality factors (e.g., perfectionism), intense external influences to become thinner, starting a sports career early in life, sport-specific training, excessive physical training, and negative coaching behaviors are potential elements that create a vulnerability for EDs in athletes (23, 24).

Regarding the role of vulnerability factors in the onset of AA, AN, and other EDs in elite athletes, several authors hypothesized that certain factors, e.g., perfectionism, doubts related to personal abilities, social uncertainty, deficits of the self-control, and idealized body image, create a fertile terrain for the development of disordered eating behaviors (25). The selection by these individuals of sports that favor a certain body weight or shape, and/or that encourage intensive physical training/constant dieting, is just an intermediary link between the vulnerability factors and the onset of EDs (25). In this context, practicing a sport that encourages a certain ideal of body shape or weight is considered a socially-acceptable framework for the manifestation (and even valorization) of disordered eating behaviors (25).

A study that explored symptomatology, personality variables associated with AN (e.g., harm avoidance, perfectionism, and obsessiveness), and lifetime EDs prevalence in Israeli women presenting AN (*N* = 31), women practicing esthetic (*N* = 111) or non-esthetic (*N* = 68) athletism, and controls (*N* = 248), found that the first group differed from the other three on all variables (26). Women in the group of esthetic athletes did not differ from the control group on any self-reported variable, but the lifetime prevalence of an eating disorder not otherwise specified was present significantly more in this group than in non-esthetic athletes or in the controls (26). Therefore, care should be given to a subgroup of female esthetic athletes who may present sub-clinical EDs for which they do not receive adequate treatment (26).

Leptin levels were lower in individuals with AA and those with AN (27). Also, delays in menarche and slower bone maturation were reported in these populations (related to low estrogen levels) (27). Therefore, by comparing the biological profiles of AA and AN patients, several authors considered that there is a common pathophysiological mechanism for the dysregulation of the reproductive axis in these two EDs (27). The exploration of salivary adiponectin can also be of use in AA patients, as in elite rhythmic gymnasts the levels of this hormone have been associated with the intensity of training and the deterioration of energy balance (28). Adiponectin levels in saliva were not, however, associated with training stress, and did not have a predictive value for reproductive dysfunction or bone mass acquisition (28).

Rhythmic gymnasts presented a risk for AN and AA according to a study that enrolled 12 members of the Norwegian national team (29). Two gymnasts had AN and two AA in this study, and all participants were dieting although they were extremely lean (29). Also, the avoidance of maturity, menstrual irregularity, energy deficit, high training schedule, and high rate of injuries were commonly met in this sample (29).

Athletes were more likely to have certain behavioral and psychological correlates of EDs when compared to non-athletes, according to a study that compared 100 female athletes and 112 non-athletes (30). Athletes also presented more frequently pathogenic weight control techniques (30).

A higher percentage of athletes competing in leanness sports was considered to have a risk of “female athlete triad” when compared to athletes competing in non-leanness sports, and more athletes competing in esthetic sports were at risk for the same pathology vs. ball game sports (31).

### 2.2. Explored criteria for identifying AA and differentiating it from similar conditions

The positive identification of this pathology is not based on specific criteria potentially due to limited epidemiological data and clinical research. However, several authors have suggested different sets of criteria, and these may be helpful in differentiating AA from other EDs or related disorders. The use of structured clinical instruments is considered useful for any ED, AA included, because they can be used for screening vulnerable populations or for monitoring the efficacy of a therapeutic intervention. This structured approach is especially important in conditions where the insight may fluctuate, as it is often the case for AA or AN (32, 33).

#### 2.2.1. Defining AA

Between the most supported symptoms and features of AA is body loss secondary to concerns related to sports activities or to the need to obtain certain results in athletic competitions, and not to the perceived deficits in body shape or general physical aspect (5). The onset of problematic behavior, defined as restrictive feeding and/or excessive exercising was determined by the need to reach certain athletic objectives or by the recommendations of the coaching team integrated into the professional training (5). There are also cyclic variations of body weight, but in some cases, the low BMI may persist even after the short-term objectives (e.g., a competition or a certain target) were reached (23). The duration of clinical manifestation is limited to the athletic career and the individual did not engage in restrictive eating or excessive exercising after that point (5).

Other authors suggest more specific criteria, like an Eating Attitudes Test (EAT-26) score ≥ 20 reflecting a preoccupation with food, calories, body shape, and weight; a score ≥ 14 on the “Body Image Dissatisfaction” scale of the Eating Disorder Inventory (EDI); an intense fear of gaining weight, even in the presence of a low BMI or extremely low body fat; the athlete preserves a body weight below the normal value for age and height, with 5–15% variations, during ≥1-year duration; restricted energy intake, severe limitation of food choice, and/or excessive exercise, i.e., more than needed for attaining good performances in sports activities; absence of medical diseases or psychiatric disorders that could explain weight loss (15). Associated features may be gastrointestinal symptoms, menstrual irregularity, frequent use of purging, or binge episodes (15).

A study that included female patients diagnosed with AN (*N* = 7), AA (*N* = 43), or BN (*N* = 42) and healthy controls (*N* = 30), compared the energy and nutrient intake for 3 consecutive days (34). A significant percentage of individuals with AN and AA had diets too low in energy and nutrients compared to the recommended levels for active females (34). The mean level of carbohydrates was lower vs. controls and protein intake was also lower than the recommended level for athletes (34). Low intake of calcium, vitamin D, and iron was also noted in these patients (34).

Age at menarche occurred later in elite athletes vs. controls, and primary amenorrhoea had a higher prevalence in the same population of professional athletes (35). Sports that emphasize thinness or a specific weight were associated with menstrual dysfunctions more than sports focused on these aspects and more than controls (35). Therefore, the significance of menstrual irregularities in this population should be interpreted with care, since it may not always be accompanied by other signs of AA.

#### 2.2.2. Differentiating AA

Anorexia athletica should be differentiated from *AN*, *BN*, and *organic pathologies*, like celiac disease, anemia, lymphoma, or bowel adenocarcinoma (34, 36).

In an analysis comparing the criteria for different EDs, restriction of calorie intake, or maintenance of a special diet was considered more important for AA than AN, and not relevant for BN; the reduction of the overall food intake and the weight loss of more than 15% were more significant for patients with AN; body image disorder, comorbid depressive features, and dysfunctional mechanisms for preservation of self-esteem were also more characteristics for patients with AN; obsessive personality features were more frequently associated with AN, while perfectionistic features with AA (37).

Regarding the differences between AA and various organic diseases, a thorough check-up containing usual blood analyses and food intolerance-specific panels should be taken into consideration. For example, in an elite female athlete, who was practicing volleyball, the main symptoms were weight loss, diarrhea, and fatigue after preseason training (30). The absence of psychological symptoms supporting an eating disorder, after careful evaluation from an interdisciplinary team, combined with a high platelet count and a duodenal biopsy, led to the formulation of a celiac disease diagnosis (36).

*Orthorexia nervosa* is another condition that should be considered when AA is suspected because these two pathologies share a common restrictive pattern of eating. Orthorexia nervosa is defined by an obsessive focus on specific foods, considered by the consumer as “healthy” which leads to significant consequences on physical, social, or psychological health (38–40).

#### 2.2.3. Structured evaluation of AA

A screening instrument was created by the Female Athlete Triad Coalition for the detection of EDs in this specific population (36, 37). This self-administered questionnaire contains three dimensions—“disordered eating,” “menstrual dysfunction,” and “skeletal health” (41, 42).

Also, a structured medical evaluation algorithm has been suggested for the at-risk population, including medical history, menstrual history, skeletal health, family/psychosocial history, physical examination, and laboratory assessments (13). Laboratory evaluations have been proposed in conjunction with a structured medical evaluation, and a complete blood cell count, metabolic parameters, erythrocyte sedimentation rate, tests for evaluation of thyroid function, vitamin D and calcium concentrations, and urine tests must be included in the initial evaluation (13).

### 2.3. Epidemiology

Elite athletes have more frequently disordered eating than the general population and non-professional sports performers (5). The difference between athletes and non-athletes was significant (13.5 vs. 4.6%), with more frequent diagnoses or isolated manifestations of EDs, and the number of cases was higher in women in a large study with Norwegian participants (21). The prevalence of EDs was evaluated in ballet dancers (*N* = 52, age 13–20) vs. patients presenting AN (*N* = 52) and non-athletic individuals (*N* = 44) of the same age, and AA was twice more frequent in the ballet professionals vs. controls (4.8 vs. 2.3%) (20).

Older studies indicate a 4–19% prevalence of EDs (AN, AA, or BN) in female college students (43). A strong emphasis on weight and a significant tendency toward EDs were reported by 6% of the non-athletes, a three times higher percentage of the athletes engaged in activities emphasizing leanness, and in one out of 10 athletes in the group, including all subdomains of athletism (43).

### 2.4. Treatment

The therapeutic management of AA involves a multidisciplinary team, with the participation of a nutritionist, an internal medicine specialist, and a psychiatrist or clinical psychologist (13). The case manager may recruit family members, coaches, and physical therapists (13). Nutritional therapy, individual cognitive-behavioral therapy (CBT), family therapy, or psychological counseling may be beneficial (13). Additional members of the team could be certified athletic trainers, exercise physiologists, the athlete’s coach, and parents or other family members (12).

No controlled study exploring the effects of medications on AA individuals has been identified in the literature.

According to the American College of Sports Medicine, “all individuals working with physically active girls and women should be educated about the Female Athlete Triad and develop plans to prevent, recognize, treat, and reduce its risks” (11). Also, programs focused on educating athletes, parents, coaches, trainers, judges, and administrators are considered a priority (12).

The first objective of the treatment is to increase energy availability by augmenting energy intake or reducing energy expenditure (12). Nutritional counseling and monitoring can usually be sufficient interventions, but more complex therapeutic strategies are granted for severe cases (12). Energy intake should be increased by 300–600 kcal/day, physical exercise should be decreased, and concomitant eating disorders require specialized treatment (44). An increase in calcium intake and vitamin D is important; in the presence of low BMI, athletes should participate in 2–3 days per week of high-impact loading and resistance training (44).

A 1-year school-based intervention program focused on the prevention of new cases of EDs among adolescent female and male elite athletes (*N* = 465) showed good results, by reducing the relative risk for current dieting and ≥ 3 weight loss attempts in the study group (45). The risk of reporting new symptoms of ED was lower in the intervention vs. control group after 1 year (45). The intervention was based on the social-cognitive framework and enhancing self-esteem by working on self-efficacy evaluations (45).

Screening programs should target evidence-based risk factors, i.e., dietary restriction, current or history of disordered eating, low BMI or recent weight loss, delayed menarche, history of current amenorrhea or oligomenorrhea, history of fractures, including multiple low-risk fractures, or single high-risk fracture (44).

## 3. Discussion

**Figure 1:**
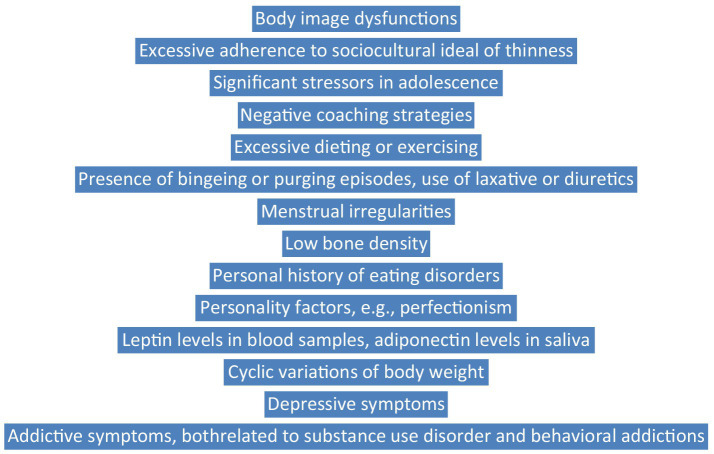
A dimensional screening model for anorexia athletica.

Data referring to the identification of AA are mostly limited to the “female athlete triad,” highlighting the presence of menstrual irregularities, bone density reduction, and disordered eating. Two sets of diagnostic criteria have been described in the literature (5, 15), but their validation through large epidemiological studies is still missing. The process of differentiating AA from similar conditions is important to include somatic investigation, in order to exclude gastrointestinal diseases or general medical conditions with negative metabolic impact. A structured evaluation is possible, and psychometric as well as screening biological procedures exist (13, 41, 42). The risk factors for EDs onset have been found, especially personality factors (e.g., perfectionism), intense external influences to become thinner, starting a sports career early in life, sport-specific training, excessive physical training, and negative coaching behaviors (23, 24), but specific factors for AA still need validation studies. Screening in vulnerable populations using structured models (Figure 1) is strongly recommended, based on key symptoms, comorbid conditions, risk factors, and biological variables. Treatment should focus on compensating metabolic deficiencies induced by prolonged dieting and psychotherapy focused on body image distortions, obsessive thoughts related to body weight or shape, comorbid mood symptoms, etc.

The results of the present article are supported by similar research in the field of EDs in athletes. For example, a narrative review concluded that screening for disordered eating behaviors and EDs should represent “a standard component of pre-participation examinations” in male and female athletes, and this would require well-informed team physicians (46). Also, the need to support the formation of “experienced multi-disciplinary teams” that should evaluate athletes with EDs was signaled by previous research (46). Prophylactic strategies for EDs are expected to include the participation of “athletes, coaches, parents, and athletic administrators” (46). A “position statement” from the Australian Institute of Sport and National Eating Disorders Collaboration shows that disordered eating is more frequent than specific EDs in athletes, which highlights the importance of early detection of sub-syndromal types of eating pathology (47). The guidelines dedicated to this topic should support “the prevention and early identification of disordered eating, and promote timely intervention to optimize nutrition for performance in a safe, supported, purposeful and individualized manner” (47). Another “position statement,” originating from the United States National Athletic Trainer’s Association, recommend the interdisciplinary approach for athlete evaluation, including specialists in “medicine, nutrition, mental health, athletic training, and athletics, in order to facilitate early detection and treatment” of disordered eating (48).

**Figure 2:**
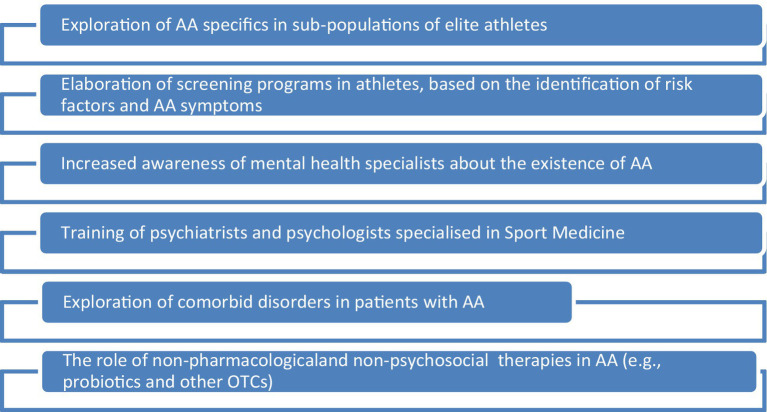
The main future directions of research in the domain of anorexia athletica. AA, anorexia athletica; OTC, over-the-counter medicines.

Future directions of research should include exploring the risk of AA in various sub-populations of elite athletes, i.e., esthetic vs. non-esthetic sports, individuals presenting a history of ED vs. those without such a history, and those practicing individual sports vs. team sports (Figure 2). Also, studies exploring the existence of AA in male athletes are very few, and the existence of gender-related particularities of this pathology requires investigation. Screening programs and early interventions designed for this population are also needed, especially since the available studies highlight the existence of AA as a subclinical type of ED. Increasing the awareness of mental health specialists about the characteristics of AA is another desideratum of a comprehensive policy targeting the improvement of quality of life in elite athletes. Also, the exploration of comorbidities in athletes with AA is granted and, due to the limitation regarding the administration of pharmacological agents in athletes, the exploration of non-pharmacological therapies may be of interest, e.g., food supplements, psychobiotics with a role in gut microbiota modulation, etc. (49–52). The role of psychotherapy for EDs, and in particular for AA, should be explored more thoroughly, using a structured, prospective approach, starting from the preliminary observations that mindfulness-based interventions (e.g., targeting mindfulness processes related to describing, expressing, and accepting emotions or discomfort when eating with others, and reducing the hyper-focus on food and body-related sensations) and CBT (e.g., cognitive restructuring, scheduling activities, behavioral exposure, and response prevention in case of excessive exercising) may be beneficial for this pathology (53–55).

## 4. Conclusion

The periodic evaluation of elite athletes, especially of those practicing esthetic sports, from an interdisciplinary perspective, using psychological and biological measures, could be helpful in the early detection of AA. Since “sports psychiatry” is a rather new branch of Medicine (56, 57), and few physicians are specialized in identifying and treating mental health dysfunctions in athletes, it is important for the coaches to involve in their teams, besides nutritionists and therapeutic physicists, mental health specialists with expertise in EDs and related health problems.

## Data availability statement

The original contributions presented in the study are included in the article/supplementary material, further inquiries can be directed to the corresponding author.

## Author contributions

OV took the entire responsibility for collecting, processing, and presenting the data herein.

## Conflict of interest

The author declares that the research was conducted in the absence of any commercial or financial relationships that could be construed as a potential conflict of interest.

## Publisher’s note

All claims expressed in this article are solely those of the authors and do not necessarily represent those of their affiliated organizations, or those of the publisher, the editors and the reviewers. Any product that may be evaluated in this article, or claim that may be made by its manufacturer, is not guaranteed or endorsed by the publisher.
